# Studies on the therapeutic effect of propolis along with standard antibacterial drug in *Salmonella enterica* serovar Typhimurium infected BALB/c mice

**DOI:** 10.1186/s12906-016-1474-5

**Published:** 2016-11-25

**Authors:** Preeti Kalia, Neelima R. Kumar, Kusum Harjai

**Affiliations:** 1 Department of Zoology, Panjab University, Chandigarh, India; 2 Department of Microbiology, Panjab University, Chandigarh, India

**Keywords:** Synergy, Antibacterial, Typhoid, Propolis, Cefixime, *Salmonella*

## Abstract

**Background:**

Antibiotic resistance is an emerging public health problem. Centers for Disease Control and Prevention (CDC) has described antibiotic resistance as one of the world’s most pressing health problems in 21^st^ century. WHO rated antibiotic resistance as “one of the three greatest threats to human health”. One important strategy employed to overcome this resistance is the use of combination of drugs. Many plants, natural extracts have been shown to exhibit synergistic response with standard drugs against microorganisms. The present study focused on the antibacterial potential of propolis in combination with the standard antibiotic Cefixime against the typhoid causing bacteria i.e. *Salmonella*.

**Methods:**

Ethanolic extract of propolis was taken for the present work. For the experiment BALB/c mice were taken as animal model and divided into ten groups. Along with normal and infected control groups, four different combinations of cefixime and propolis were used. Biochemical, hematological and histopathological indices were studied by following the standard protocols.

**Results:**

In BALB/c mice, *Salmonella* causes severe biochemical, hematological and histopathological alterations by 5^th^ day of infection. Ethanolic extract of propolis at a dose of 300 mg/kg body weight of mice when used alone to treat *Salmonella* infection in mice gave significant results by 30^th^ day of treatment. Similarly, when cefixime (4 mg/kg body weight of mice) was used to treat infection in mice, significant results as compared to infected control were observed after 5^th^ day. But when propolis and cefixime were used together in different concentrations in combination therapy, evident results were observed after 5 days of treatment. The levels of various liver and kidney function enzymes, blood indices and the histopathology of liver, spleen and kidney were restored to near normal after 5 days of treatment and at much lower doses as compared to the effective dose when used alone.

**Conclusion:**

The study confirmed that significant results were observed in three combinations of cefixime and propolis as compared to infected controls. Propolis acted synergistically with cefixime and enhanced the efficacy of antibiotic and reduced its effective dose in combined therapy.

## Background

*Salmonella* are Gram negative bacteria that have gained importance not only as a threat to public health worldwide but also as a model for studying the mechanisms of bacterial pathogenesis. Primarily two serotypes of *Salmonella enterica* i.e. Typhi and Paratyphi are responsible for causing typhoid fever in India [1]. Typhoid fever and gastroenteritis are the principal clinical syndromes associated with *Salmonella*. Typhoid occurs in various parts of the world like Asia, South America, Middle East, Zimbabwe, India, Florida, Spain, Thailand, Turkey, Nigeria and many more [2, 3].

Every year around 21 million cases of typhoid fever are reported worldwide [4]. The identified risk factors include intake of street food, ice creams, contaminated water, poor sanitary conditions at home and excessive use of antimicrobial drugs [5]. The commonly used therapy against typhoid is the use of antimicrobials like some fluoroquinolones, cephalosporins, azalides. One such cephalosporin is Cefixime which acts by penetrating into the monocytes and causes morphological changes and growth inhibition of *S. typhimurium*. Because of increasing multi drug resistance (MDR) there is a worrisome possibility of recurrence of untreatable typhoid [6]. Studies favoring the use of different biologically active natural products for the treatment of serious ailments are now being emphasized.

Honey bee propolis has attracted the interest of scientists and researchers on account of its remarkable pharmacological and biological properties. It is a very sticky, resinous substance which is collected by honey bees from the barks and buds of plants. It is mixed with saliva, some enzymes and used by bees to seal the walls of hive to ensure a hygienic environment [7]. Propolis acts as a “Chemical Weapon” for the bees protecting them from the attack of small insects and microorganisms. Bees set an example of maintaining such a naturally sterile home. Research has been done to demonstrate the antibacterial, antifungal, antiviral, antioxidant and other biological activities of propolis [8]. Various clinical studies are in progress to verify the preventive and therapeutic potential of propolis as an antibiotic alone as well as synergistically.

*Salmonella* species cause infection ranging from asymptomatic carriage and localized gastroenteritis to systemic enteric fever [9]. *Salmonella enterica* serovar Typhimurium causes an invasive disease in mice that has similarity with human typhoid. Moreover, animals are the best suited models for the development or authentication of any new drug or the characterization of a new drug. Out of different strains of mice, BALB/c strain is the most susceptible mouse lineage to study the pathogenesis of the disease. Mutated Nramp 1 gene is responsible for the susceptibility [10]. Mice having typhoid showed signs like elevated temperature, ruffled fur, lethargic behavior. Therefore, BALB/c mice were selected as animal model for the present study.

The present study is a step to support the use of propolis as a “global remedy”. This study aims to evaluate propolis as a potential strategy to fight the problem of MDR by using it in a combination therapy whereby two drugs are used together against a disease. This therapy helps to reduce the dose of the synthetic antimicrobial if the second component is an effective natural product that has ameliorative/curative properties. Plants and other natural sources are being extensively exploited in this direction.

## Methods

### Collection of propolis and preparation of extracts

Propolis was obtained from honey bee hives kept in an apiary maintained by Department of Zoology, Panjab University, Chandigarh, India. Ethanolic extract was prepared by following standard protocol as it gave the best results in previous studies [8]. 30 g of propolis was ground and 70% ethanol was added to make a total volume of 100 mL. The components were mixed, kept away from bright light, at room temperature and with moderate shaking. After 5 to7 days the solution was filtered and dried. Propolis was stored in a dry and cool place (freezer) after extraction. The percentage yield of propolis was calculated by the formula:

$$ \mathrm{Percentage}\ \mathrm{yield}\ \left(\%\right)=\frac{Amount\  of\  pure\  product\  recovered}{Amount\  of\  crude\  material\  used}\times 100 $$

Specific dilutions of propolis were then prepared as required.

### Microorganism

The bacterial strain of *Salmonella enterica* serovar Typhimurium (MTCC 98) was procured from IMTECH, Sector-39, Chandigarh and stored in the form of small aliquots at −20 °C before subculturing. The strain was examined biochemically before storage and use.

### Experimental animal model

White swiss mice of BALB/c strain (weighing 25–28 g and 4–6 weeks old) of either sex were used as experimental model. Mice were obtained from Central Animal House, Panjab University, Chandigarh. The animals were maintained in lab in animal house of Panjab University at 25 ± 2 °C temperature under 12 hr light/dark cycle. The treatment of the mice was according to the guidelines of committee for the purpose of control and supervision of experiments on animals and was approved by Institutional Animal Ethics Committee (IAEC/411 dated 11/9/2013) of Panjab University, Chandigarh. All the animals were housed in polypropylene cages and fed with a standard pellet diet (Ashirwad industries, Punjab, Hindustan Lever, India) and water *ad libitum*.

### Study design

Mice were segregated into ten groups, with 6 mice in each group as follows: Gp **Normal:** Normal mice given saline orally (negative control). Gp **Infected:** *Salmonella enterica* serovar Typhimurium infected: Mice given 2X10^4^ CFU/mL of *Salmonella* (0.2 mL) once *i.p*. (positive control). Gp **OC**: Only cefixime [4 mg/kg body weight of mice (b.w.)] treated groups without infection for 5 days. Gp **OP**: Only propolis (300 mg/kg b.w.) treated groups without infection for 30 days. Gp **C**: *Salmonella* infected + 4 mg Cefixime /kg b.w.: Mice were given antibiotic cefixime for 5 days (orally). Gp **P**: *Salmonella* infected + propolis at the dose of 300 mg/kg b.w. for 30 days. Gp **CP1:** *Salmonella* infected + Combination of cefixime and propolis (3 mg/kg b.w. + 225 mg/kg b.w.) respectively given orally. Gp **CP2:** *Salmonella* infected + Combination of cefixime and propolis (3 mg/kg b.w +150 mg/kg b.w.). Gp **CP3:** *Salmonella* infected + Combination of cefixime and propolis (2 mg/kg b.w. + 225 mg/kg b.w.). Gp **CP4:** *Salmonella* infected + Combination of cefixime and propolis (2 mg/kg b.w. + 150 mg/kg b.w.). Mice were given combination for 5 days orally after infection. Animal sacrifices were made after the completion of treatment.

### Bacterial load

The bacterial loads in blood, liver, spleen and kidney of mice were determined by following the standard protocols [11].

### Biochemical studies

Blood was aspirated from jugular vein of mice of different experimental groups and kept at room temperature for 20 mins. It was then centrifuged at 3000 rpm for 10 mins. to obtain the serum. All the marker tests were performed by using commercially available kits.

### Hematological studies

Appropriate quantities of blood samples were collected from the jugular vein of mice in sodium salt of ethylenediaminetetraacetic acid (EDTA) for estimation of red blood cell count (RBC), hemoglobin concentration (Hb), packed cell volume (PCV), total leucocyte count (TLC). Mean corpuscular volume (MCV), mean corpuscular hemoglobin (MCH) and mean corpuscular hemoglobin concentration (MCHC) were also calculated [12].

### Histopathological studies

In order to investigate pathological changes in the selected tissues of experimental animals, histological studies were performed. For these studies animals were dissected and different tissues like liver, kidney and spleen were taken from normal, infected and treated mice. The tissues were rinsed in normal saline, weighed, fixed and then further processed by following the standard protocol [13, 14].

### Statistical analysis

Data were expressed as mean ± S.D. All experiments were repeated thrice. The statistical significance of inter group difference of biochemical parameters and microbial counts was determined by Analysis of Variance (ANOVA) using Tukey test. Differences were considered statistically significant at *p* < 0.05 and highly significant at *p* < 0.001.

## Results

### Yield of propolis extract

The principal solvents used for extraction of bioactive compounds from crude propolis (30 g) was ethanol. The weight of propolis extracted and percentage yield from ethanol was 7.019 g and 57.2% respectively.

This present study was an attempt to test propolis, a natural product of the bee hive, for its antimicrobial effect in combination with cefixime against *Salmonella enterica* serovar Typhimurium. Concentrations used in combination were decided on the basis of best results obtained in monotherapy experiments. The best dose obtained in monotherapy experiments was 300 mg/kg b.w. of propolis [9]. Concentrations of propolis used for present experiments were ¾ and ½ of the best dose i.e. 225 mg and 150 mg respectively. Similarly the concentration of cefixime for combination experiments were ¾ and ½ of 4 mg i.e. 3 mg and 2 mg respectively.

### Survival percentage

The number of animals that survived after treatment with different combinations of propolis and cefixime substantiates the aim of study. In the infected group (without treatment) 94.94 ± 9.62% survival was recorded on 5^th^ day of infection. However animals presented signs of weakness, lean body, lethargic behavior and hunched back. Fifth day was the peak day of infection as revealed by the analysis of bacterial count in blood during the experiment. After 5^th^ day no survival was observed in Infected group. In combination treatment groups 100% survival was recorded in CP1, CP2 and CP3 group whereas in case CP4, the survival percentage was the same as infected mice after 5 days of treatment (Table 1). Since in case of propolis treated group (P), effective results were seen by 30^th^ day of propolis treatment so the survival percentage was recorded till last day of treatment i.e. 30^th^ day. In the follow up study (30 days after the end of treatment), survival percentage of all groups was recorded.

**Table 1:** Percentage survival of animals

Groups	Percentage (%) Survival of animals		
	At start of treatment	End of treatment	Follow up
Normal	100 ± 0	100 ± 0	100 ± 0
Infected	100 ± 0	94.94 ± 9.62	NS
OC	100 ± 0	100 ± 0	100 ± 0
OP	100 ± 0	100 ± 0	100 ± 0
C	100 ± 0	100 ± 0	100 ± 0
P	100 ± 0	77.53 ± 9.98	77.53 ± 9.98
CP1	100 ± 0	100 ± 0	100 ± 0
CP2	100 ± 0	100 ± 0	100 ± 0
CP3	100 ± 0	100 ± 0	NS
CP4	100 ± 0	94.94 ± 9.62	NS

Data is expressed as mean ± SD (*n* = 6), *NS*: No mice survived

### Bacterial load in blood and different organs

These studies were conducted over a period of five days.

Blood: The bacterial count in the blood was observed to be 6.57 ± 0.15 log CFU/mL at 120 hrs. after infection. In case of only cefixime and only propolis treated groups significant decline in count was observed. On treating with different combinations of propolis and cefixime it was found that in case of CP1, CP2 and CP3 there was significant decrease in bacterial count (Table 2).

**Table 2:** Bacterial load in blood of mice after *Salmonella enterica* serovar Typhimurium infection and during the course of treatment in combination therapy

Time	0–4 h	4–8 h	8–12 h	12–24 h	72 h	120 h
Groups						
Infected	4.16 ± 0.14	5.46 ± 0.07	4.57 ± 0.06	4.47 ± 0.1	5.34 ± 0.14	6.57 ± 0.15
C	4.25 ± 0.15	4.86 ± 0.19	3.64 ± 0.3	3.07 ± 0.005	2.57 ± 0.2^#^	0^#^^
P	4.27 ± 0.18	4.34 ± 0.25	4.21 ± 0.31	4.12 ± 0.17	3.5 ± 0.46	3.36 ± 0.25^#@^
CP1	4.34 ± 0.15	4.5 ± 0.19	4.1 ± 0.30	3.36 ± 0.005	3.51 ± 0.2	2.15 ± 0^#@^
CP2	4.57 ± 0.11	4.34 ± 0.26	4.21 ± 0.18	4.12 ± 0.48	3.5 ± 0.19	3.12 ± 0.18^#@^
CP3	4.27 ± 0.18	4.34 ± 0.25	4.21 ± 0.31	4.12 ± 0.17	3.5 ± 0.46	3.36 ± 0.25^#@^
CP4	4.54 ± 0.06	5.53 ± 0.39	5.86 ± 0.17	5.47 ± 0.19	4.34 ± 0.06	4.24 ± 0.13^@^

Data is expressed as mean **±** SD

^#^ *p* value Infected *vs* C, P, CP1, CP2, CP3 and CP4

^@^ *p*-value C vs P, CP1, CP2, CP3 and CP4

^^^ *p*-value P vs C, CP1, CP2, CP3 and CP4

(^*@^^ *p* < 0.05: statistically significant), (^#^ *p* < 0.001: statistically very significant)

**Fig. 1:**
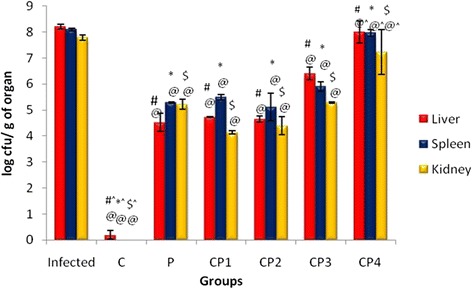
Histogram showing the bacterial load in different organs of mice after *Salmonella enterica* serovar Typhimurium infection and treatment in combination groups. Data is expressed as mean **±** SD. ^#^ *p*-value Infected liver *vs* treated liver, ^*^ *p*-value Infected spleen *vs* treated spleen. ^$^ *p*-value Infected kidney *vs* treated kidney. ^@^ *p*-value C vs P, CP1, CP2, CP3 and CP4. ^^^ *p*-value P vs C, CP1, CP2, CP3 and CP4. (^@^^ *p* < 0.05), (^#*$^ *p* < 0.001)

In case of bacterial load in organs like liver, spleen and kidney again significant decline in bacterial load as compared to infected control was observed in CP1, CP2 and CP3 group (Fig. 1).

### Biochemical studies

The levels and activities of various enzymes to assess the functioning of liver and kidney were estimated in serum of different groups of mice.

#### Liver and kidney function tests

The estimation of Alanine aminotransferase (ALT)/ serum glutamate pyruvate transaminase (SGPT), Aspartate aminotransferase (AST)/ serum glutamate oxaloacetate transaminase (SGOT), alkaline phosphatase (ALP), bilirubin was done in serum samples by using commercially available kits. The levels of AST, ALT, bilirubin and ALP were increased in infected group as compared to normal group. On giving treatment of combination of cefixime and propolis significant decrease was observed in CP1, CP2, CP3 groups, showing the effectiveness of these combination. Similarly in case of kidney function tests the level of urea, uric acid, creatinine and uric acid was increased in case of infected control but a significant decline was observed in combination 1, 2 and 3 (Table 3).

**Table 3:** Results of liver, kidney function tests and levels of protein and glucose in the serum of experimental groups

Group	ALT (IU/L)	AST (IU/L)	ALP (KA units)	Bilirubin (mg/dL)	Urea (mg/dL)	BUN (mg/dL)	Uric Acid (mg/dL)	Creatinine (mg/dL)	T. Protein (g/dL)	Glucose (mg/dL)
Normal	21.4 ± 0.87	25.97 ± 1.04	8.57 ± 0.73	0.85 ± 0.12	43.26 ± 0.9	19.89 ± 0.41	3.03 ± 0.2	0.38 ± 0.056	6.93 ± 0.23	133.33 ± 6.67
Infected	140.57 ± 1.27^$^	94.03 ± 3.5^$^	28.33 ± 1.52^$^	2.2 ± 0.17^$^	82.76 ± 5.68^$^	38.07 ± 2.6^$^	6.26 ± 0.25^$^	0.78 ± 0.02^$^	5.2 ± 0.4^$^	108.33 ± 3.85^$^
OC	19.76 ± 0.60	25.5 ± 1.44	8.66 ± 0.57	0.72 ± 0.035	44.5 ± 1.80	20.47 ± 0.82	4.93 ± 0.11	0.34 ± 0.02	6.95 ± 0.23	135.56 ± 3.85
OP	35.43 ± 4.9	19.66 ± 0.57	7.66 ± 0.5	0.74 ± 0.04	43.86 ± 1.62	20.49 ± 0.88	3 ± 0.1	0.3 ± 0.02	6.533 ± 0.23	135.55 ± 3.85
C	23.3 ± 2.23^#^	26.44 ± 3.23#	8.77 ± 0.36^#^	0.66 ± 0.06^#^	43.86 ± 1.62^#^	20.49 ± 0.88^#^	3 ± 0.1^#^	0.34 ± 0.01^#^	6.76 ± 0.05^#^	135.55 ± 3.85^#^
P	51.46 ± 3.1^#^	35.43 ± 4.9^#^	15.2 ± 2.85^#^	0.7 ± 0.04^#^	52.7 ± 2.94^#^	24.23 ± 1.35^#^	4.24 ± 0.41^#^	0.41 ± 0.02^#^	7.06 ± 0.23^#^	131.1 ± 3.85^#^
CP1	22.33 ± 0.57^#^^	27.33 ± 3.05^#^	8.72 ± 0.42^#^	0.66 ± 0.06^#^	44.5 ± 1.8^#^	20.47 ± 0.82^#^	3.03 ± 0.05^#^	0.3 ± 0.01^#^	6.77 ± 0.04^#^	137.77 ± 3.85^#^
CP2	23.3 ± 2.23^#^^	33.32 ± 2.88 ^#^	10.33 ± 2.88^#^	0.66 ± 0.02^*@^	43.96 ± 1.76^#^^	20.22 ± 0.81^#^^	3.16 ± 0.15^#@^	0.39 ± 0.04^#^	7.06 ± 0.23^#^	133.11 ± 7^#^
CP3	88.57 ± 3.9^#@^^	51.73 ± 2.82 ^#@^^	17.86 ± 1.76^#@^	0.83 ± 0.03^#^	65.3 ± 4.65^#@^^	30.03 ± 2.14^#@^^	5.2 ± 0.43^@^	0.69 ± 0.01^*@^^	5.86 ± 0.46^@^^	111.1 ± 3.85^@^^
CP4	139.67 ± 0.58^@^^	93.1 ± 2.07^@^^	27.33 ± 0.57^@^^	2.1 ± 0.17^@^^	79.66 ± 0.57^@^^	36.64 ± 0.26^@^^	5.9 ± 0.17^@^^	0.71 ± 0.02^@^^	5.06 ± 0.23^@^^	108.88 ± 3.85^@^^

Data is expressed as mean **±** SD. (ANOVA: Tukey test)

^$^ *p*-value Normal *vs* Infected, OC, OP (*p* < 0.001: statistically very significant)

^*#^ *p*-value Infected *vs* C, P, CP1, CP2, CP3, CP4

^@^ *p*-value C vs P, CP1, CP2, CP3 and CP4

^^^ *p*-value P vs C, CP1, CP2, CP3 and CP4

(^*@^^ *p* < 0.05: statistically significant), (^$#^ *p* < 0.001: statistically very significant)

### Hematological studies

Hematological indices provide very important information regarding the well being of an individual. The results showed alterations in infected control as compared to normal ones. After treatment with different combinations of cefixime and propolis the values were restored near normal (Table 4).

**Table 4:** Results of hematological indices in the blood of experimental groups

Group	RBC (million/mm3)	TLC/mm^3^	Hb (g/dL)	PCV (%)	MCV μm3	MCH (pg)	MCHC (%)
Normal	8.36 ± 1.2	7730 ± 170.88	12.48 ± 0.66	42 ± 1	48.83 ± 3.85	13.68 ± 1.88	29.27 ± 0.82
Infected	4.37 ± 0.95^$^	5766.66 ± 28.86^$^	9.06 ± 0.11^$^	28 ± 1^$^	64.17 ± 0.14^$^	21.02 ± 0.75^$^	34.13 ± 3.89^$^
OC	6.90 ± 0.70	7669.33 ± 111.26	12.31 ± 0.15	41.66 ± 1.52	53.63 ± 5.94	17.72 ± 2.13	33.01 ± 0.50
OP	8.06 ± 0.66	7348.33 ± 47.52^$^	12.6 ± 0.55	44 ± 1.73	53.49 ± 3.79	14.82 ± 1.23	28.68 ± 1.35
C	7.76 ± 0.58^*^	7563 ± 32.14^#^	11.56 ± 0.4^#^	40.33 ± 0.57^#^	52.12 ± 3.84^*^	15.89 ± 3.26^#^	28.68 ± 1.35^#^
P	8.06 ± 0.66^*^	7348.33 ± 47.52^#^	12.6 ± 0.55^#^	44 ± 1.73^#^	53.49 ± 3.79^*^	14.82 ± 1.23^#^	28.69 ± 2.3^*^
CP1	8 ± 0.95^*^	7091.66 ± 141.8^#^	12.4 ± 0.3^#^	40.5 ± 2.45^#^	53.84 ± 6.03^*^	15.59 ± 1.58^#^	30.7 ± 2.2^*^
CP2	7.59 ± 0.45^*^	7073.33 ± 110.15^#@^^	13.5 ± 0.82^#^	40.2 ± 2.1^#^	53.2 ± 6.01^*^	17.64 ± 2.16	30.93 ± 0.05^*^
CP3	6.46 ± 0.05	6903.33 ± 41.63^#@^^	11.1 ± 0.1^*^	33.26 ± 2.58^*@^^	51.41 ± 3.55^*^	17.16 ± 0.21	33.49 ± 2.76
CP4	4.6 ± 0.69^@^^	5885 ± 186.21^@^^	10.7 ± 0.34^@^^	28.83 ± 0.57^@^^	62.9 ± 1.96	20.41 ± 1.28	33.59 ± 0.57

Data is expressed as mean **±** SD. (ANOVA : Tukey test)

^$^ *p*-value Normal *vs* Infected, OC, OP (*p* < 0.001: statistically very significant)

^*#^ *p*-value Infected *vs* C, P, CP1, CP2, CP3, CP4

^@^ *p*-value C vs P, CP1, CP2, CP3 and CP4

^^^ *p*-value P vs C, CP1, CP2, CP3 and CP4

(^*@^^ *p* < 0.05: statistically significant), (^$#^ *p* < 0.001: statistically very significant)

Along with comparison of treatment groups with infected control, the effect of cefixime and propolis was compared with different combinations to understand whether the combination was effective because of effect of cefixime. By making the comparison it was observed that most of the indices of CP1 group showed no significant difference as compared to cefixime or propolis. This suggests that the effectiveness was due to action of both the antibiotic and propolis.

### Histopathological studies

The histopathological analysis of different organs of all experimental groups was done with the aim to determine the curative effect of honey bee propolis in combination with standard antibiotic cefixime against *Salmonella enterica* serovar Typhimurium infection in BALB/c mice.

#### Liver

The transverse section of normal liver showed hepatic lobules consisting of large polygonal cells, the hepatocytes arranged radially around the central vein (Plate I: a, b and c). In case of infected control, the haematoxylin and eosin stained sections of infected liver showed prominent infiltration of lymphocytes (Plate I: d), dilated sinusoids, Kupffer cells and lymphocytes were seen in dilated sinusoids. Hepatocyte degeneration, destruction, microvesicular fatty changes, acute liver necrosis, fairly heavy lobular hepatitis (Plate I: e) and chronic portal triaditis (Plate I: f) were also observed. Formation of localized clusters of lymphocytes which resulted in formation of typical “typhoidal nodules” was observed. In cefixime treated liver of mice, no significant pathological changes were observed after the completion of treatment period and the cytoarchitecture was similar to that of normal liver with prominent hepatic cords radiating outwards from central vein. Haematoxylin and eosin stained transverse section of group P showed almost regular liver histology. The histology of liver of mice treated for 5 days with combinations revealed regular hepatic architecture. Polyhedral hepatocytes were radiating outwards from central vein. No infiltration of lymphocytes was observed in CP1, CP2 and CP3 (Plate II: a, b, c and d). In case of CP4, histology of liver included lymphocytic infiltration and vacuolization, distorted portal triad showing the ineffectiveness of this combination as compared to other treatments (Plate II: e and f).

**Plate I:**
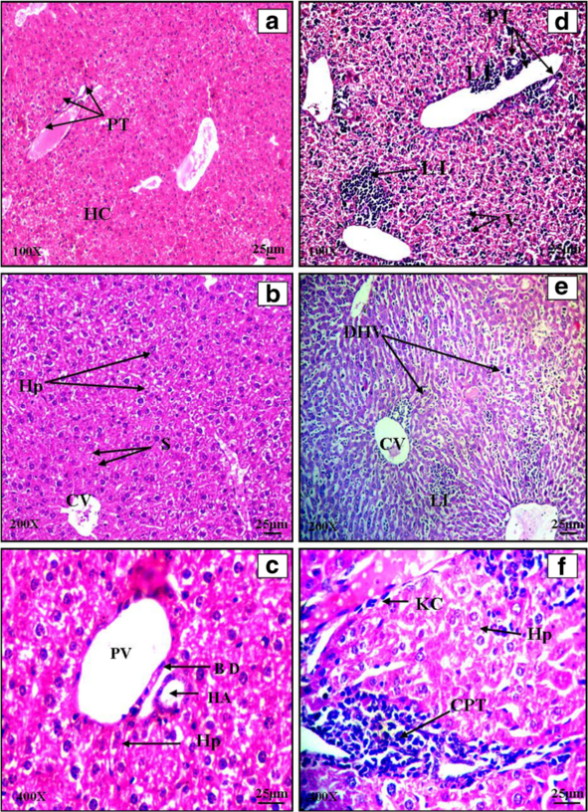
Haematoxylin and eosin stained transverse sections of liver of normal (**a**, **b**, **c**) and *Salmonella enterica* serovar Typhimurium infected BALB/c mice (**d**, **e**, **f**)

**Plate II:**
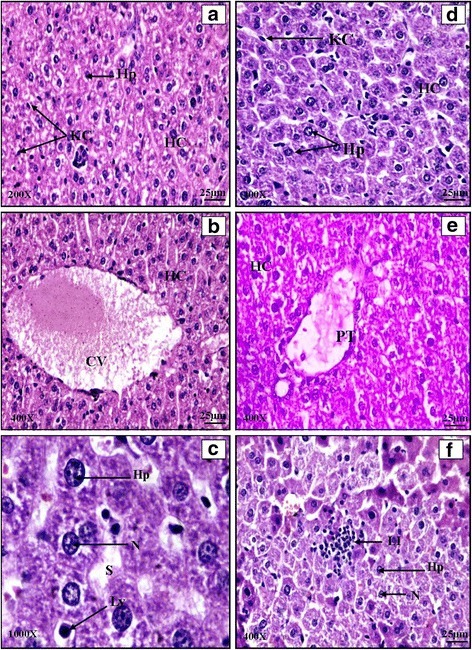
Haematoxylin and Eosin stained transverse sections of liver of combination therapy groups CP1 (**a**), CP2 (**b** and **c**), CP3 (**d**), CP4 (**e** and **f**)

#### Spleen

Spleen is a blood filter and a highly vascular organ. Light microscopic studies of spleen showed normal morphology having white and red pulp region separated by the marginal zone. (Plate III: a, b and c). Infected spleen showed changes in the form of non follicular diffused enlargement of white pulp and reduction of red pulp region commonly known as non follicular lymphoid hyperplasia. Infected spleen showed the reactive enlargement of follicles and increase in the number of follicles (Plate III: d, e and f). Transverse section of cefixime treated spleen and propolis (300 mg) treated spleen showed normal structural organization. Combination of cefixime and propolis in CP1, did not allow the alterations in spleen to occur Plate IV: a). The histology of spleen of mice treated with combined dose of cefixime and propolis (CP2, CP3) for 5 days showed normal splenic architecture. Red pulp was clearly visible. Normal white pulp having follicles, PALS and marginal zone was clear (Plate IV: b, c and d). No improvement was seen in spleen of CP4 group. Neither red nor the white pulp was clear in spleen. Hyperplasia was observed in CP group and there was complete distortion of marginal zone (Plate IV: e and f).

**Plate III:**
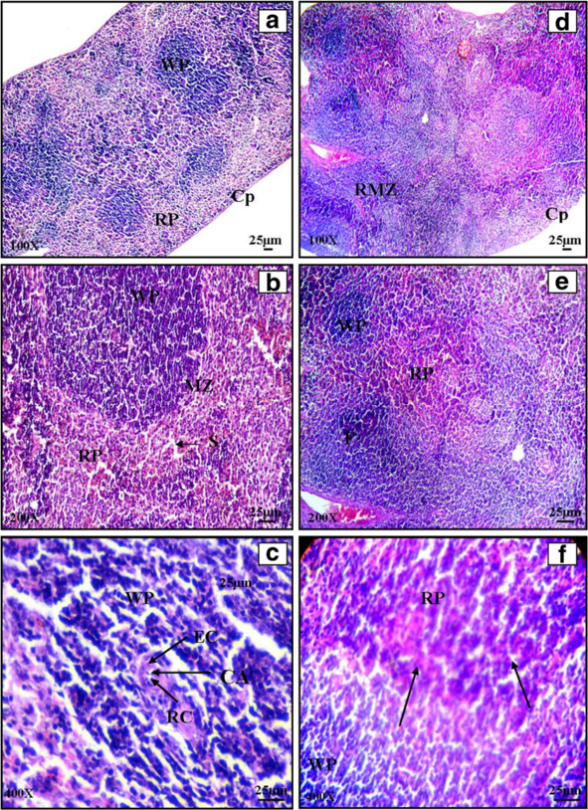
Haematoxylin and eosin stained transverse sections of spleen of normal (**a**, **b**, **c**) and *Salmonella enterica* serovar Typhimurium infected BALB/c mice (**d**, **e**, **f**)

**Plate IV:**
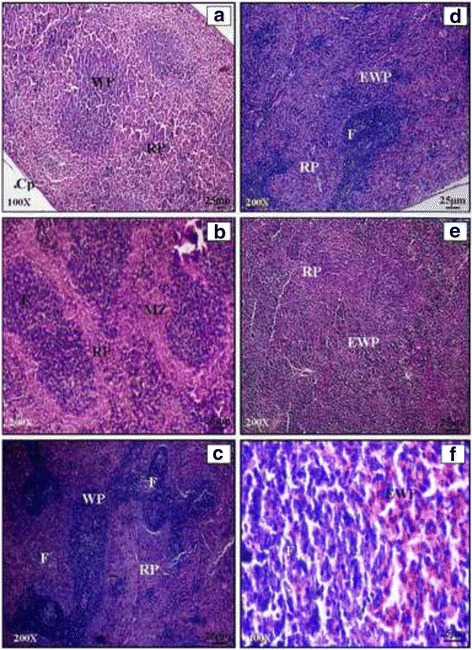
Haematoxylin and Eosin stained transverse sections of spleen of combination therapy groups CP1 (**a**), CP2 (**b**), CP3 (**c** and **d**), CP4 (**e** and **f**)

#### Kidney

Histological examination of a normal BALB/c mice kidney revealed typical general organization with outer cortex and inner medulla region. Cortex region consisted of small spherical bodies called renal corpuscles which showed 2 parts i.e. glomerulus and Bowman’s capsule. The medullary region consisted of renal pyramids (Plate V: a, b, c) The effect of *Salmonella enterica* serovar Typhimurium infection was not as severe as in the reticuloendothelial organs (liver and spleen). The architecture of infected kidney appeared normal at low magnification. At higher magnification, histology showed excessive lymphocytic infiltration on 5^th^ day of infection (Plate V: d, e, f). Histology of cefixime (C) and propolis (P) treated mice kidney revealed normal structural organization with distinguishable cortical and medullary regions. Normal renal corpuscles were evident. Proximal and distal convoluted tubules were clearly distinguishable. The histological studies of combination groups clearly indicated signs of recovery in case of CP1, CP2 and CP3 (Plate VI: a, b, c, d and e). The architecture was restored to normal showing distinct Bowman’s capsule, glomerulus, mesangial space, PCT and DCT. The kidney histology of animals in CP4 group was more like infected mice with lymphocytic infiltration in kidney (Plate VI: f). However the impact of *Salmonella* infection was more in liver and spleen as compared to kidney.

**Plate V:**
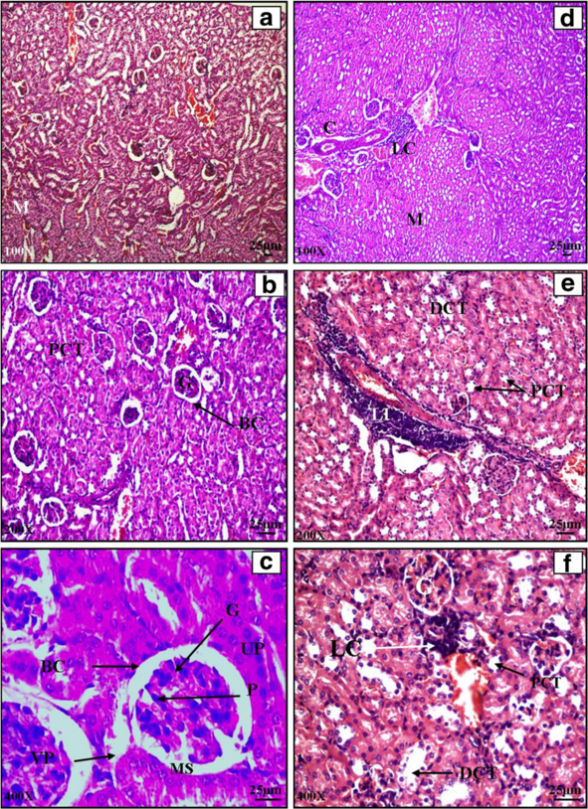
Haematoxylin and eosin stained transverse sections of kidney of normal (**a**, **b**, **c**) and *Salmonella enterica serovar Typhimurium* infected BALB/c mice (**d**, **e**, **f**)

**Plate VI:**
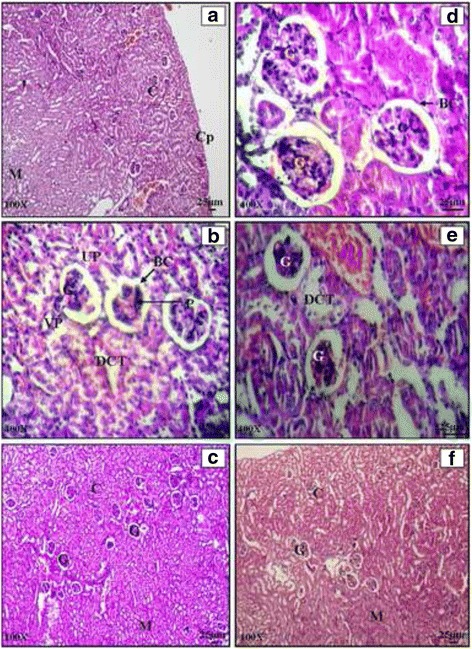
Haematoxylin and Eosin stained transverse sections of kidney of combination therapy groups CP1 (**a** and **b**), CP2 (**c** and **d**), CP3 (**e**), CP4 (**f**)

## Discussion

In an effort to improve the quality of life, man has often turned to plants as source of food, clothing, shelter and home remedies. Plants contain certain active substances that are responsible for rendering them their medicinal properties. Plants products have therefore found extensive use in traditional methods of treatment. Pharmaceutical industry produces a number of drugs with quick and lasting treatment effects. But the increasing resistance against these drugs in the present scenario has diverted the focus to some natural products or extracts which can replace or supplement modern medicine.

Survival is the first and foremost noticed factor in any experiment. In the present study 100% survival was observed in groups CP1, CP2, CP3 at the end of treatment regimen as compared to infected control. Previous studies confirmed that infection with *Salmonella enterica* serovar Typhimurium in mice caused mortality in 5–7 days [15]. The mortality could be due to severe effect of *Salmonella* infection on vital organs as well as on physiological parameters. Treatment with natural extracts like that of *Withania somnifera*, *Houttuynia cordata,* peel extract of *Punica granatum* was shown to increase the survival rate as compared to infected mice [16, 17].

As observed in the present study, the infected mice showed heavy bacterial loads in different organs. However, a significant reduction in bacterial load was observed in combination treated mice. Earlier investigations showed the use of propolis against bacteria of human infections, and also reported that lower concentrations of propolis were effective against Gram positive bacteria but higher doses had to be used against Gram negative bacteria [18]. Several authors observed that the biological activities of propolis were due to the presence of various phytochemicals [8, 19–22]. Studies confirmed that phenolic acid components present in propolis caused inhibition of microbial enzymes thus depriving the microorganism. The various biological activities of propolis including the antibacterial property were due to the presence of flavonoids, CAPE, esters as supported by the findings of many researchers. It was proved that a single component of propolis was not as effective as the total extract, which further supported that synergism existed in various compounds of propolis to make effective biological impact [23]. On the other hand, the effectiveness of cefixime was due to its reasonable penetration into the monocytes thus inhibiting the growth of bacteria [24].

In the present study, during combination trials, a synergistic behavior of propolis with cefixime was observed which further facilitated the activity of cefixime and reduced its dose. Though the exact mechanism of action of synergistic effect is not yet known, the synergy might be due to some complex formation which inhibited the microorganisms by interfering with its cell wall synthesis or by lysing the cell and thus causing death of microorganism or by making the bacterial cell wall more permeable [25]. Both cefixime and propolis complemented each other’s activity during the present study. It is known that even if a bioactive substance has little activity against causative agent, it will assist the major active component in its action against bacteria (pharmacokinetic synergy) [5]. In previous studies propolis has been used in combination with chloramphenicol, tetracycline and neomycin against *S. typhi* in vitro [26]. The results supported that ½ and ¼ MIC of propolis when used in combination with the antibiotic was able to reduce the log count of bacteria. Synergistic effect of propolis with tetracycline was also observed against *S. aureus* [27].

In the present experiment, to assess the damage caused to liver by *Salmonella*, the levels and concentrations of enzymes and macromolecules like AST, ALT, ALP, bilirubin were measured. These enzyme molecules are present in hepatocytes in normal individual. The raised levels of these in blood gives clear indication of some hepatic damage. Hepatic injury could be caused by endotoxins, non specific reactive inflammation or some cytotoxins produced by bacteria that have infected Kupffer cells [28]. Previous studies reported the hepatoprotective effect of ethanolic extract of propolis [29, 30]. Propolis showed inhibitory activity against oedema, leakage, conglomeration in case of inflammations that were caused due to microorganism infiltration or toxins or any injury [31–34]. Damage to the liver was reduced when treated with different doses of propolis in the present study. Propolis has been observed to act as a strong antioxidant agent and hence as a scavenger of free radicals [35]. It has been suggested that the hepatic preventive activity of propolis was because it suppressed the leakage of enzyme through plasma membrane of cells [36]. Propolis could also cause the repair of damaged cell wall of hepatocytes and increase their functional properties [37, 38].

The levels of some kidney enzymes were raised in infected group which were restored to near normal values after treatment with cefixime – propolis combination. Increase or decrease in some indices might be due to hampered glomerular filtration of urea and creatinine [39–41].

Blood is considered as an essential body fluid that regulates the various vital functions of the body such as transport of nutrients, gases and protection against invaders*.* Hematological indices provide very important information regarding the well being of an individual [42]. Typhoid fever causes a lot of hematological disturbances which include anemia, leucopenia, eosinophilia [43, 44]. In the present study, *Salmonella enterica* serovar Typhimurium infection significantly decreased the mean levels of RBC, Hb, PCV. According to earlier studies [45] haemophagocytosis and bone marrow suppression were responsible for hematological changes White blood cells are the cells of immune system that help in protecting the body against foreign intruders. Any alteration in the WBC count acts as an indicator of disease. Significant decrease in the WBC count in typhoid mice was observed when compared with healthy control and these results were supported by the earlier findings [46, 47] which showed leucopenia in 4% of the total typhoid cases. The results of all treatments except CP4 showed significantly different results as compared to infected control. The polyphenolic compounds present in propolis and other bee products have protective effect for the RBC cell membrane [15, 48, 49]. The components of propolis like cinnamic derivatives and some flavonoids caused the loss of membrane potential due to the increase in permeability of bacterial membrane to ions and this altered the bioenergetic state of microorganism [50].

Histological studies during the present study showed that during typhoid, reticuloendothelial organs were mainly involved [51]. Previous studies have reported that mice infected with *Salmonella enterica* serovar Typhimurium showed signs of histological damage [52]. Combination therapy in groups CP1, CP2 and CP3 was found safe on the hepatic architecture of mice. Earlier studies confirmed that with the help of treatment with propolis the multifocal nephritic changes in liver were completely reversed showing its effectiveness [53]. In case of spleen, various researchers showed multifocal histiocytic infiltration, lymphoid follicular disruption and thrombosis which resembled the pathology of people having haemophagocytic lymphohistiocytosis [54]. In kidney, the findings by various researchers [17] confirmed that *Salmonella enterica* serovar Typhimurium caused negligible architectural changes. Mice treated with propolis and cefixime both singly as well as in combination showed signs of recovery. Moreover, previous studies by authors concluded that propolis has no negative effect if consumed directly and hence no toxicity was reported till the consumption of 5 mg/kg b.w. of propolis by mice [55]. The overall results of the present work provide baseline information for the possible use of the ethanolic extract of propolis (EEP) in the treatment of salmonellosis, especially typhoid fever.

## Conclusion

From the present experiments it is clear that the combination therapy of cefixime and propolis (CP1 group) is most effective against *Salmonella* infection as it not only reduced the doses of the two components but also showed significant results after five days of treatment which was also supported by the follow up studies. However, more studies are required to test the combination in higher animal models before it is applied in typhoid patients. The challenge to look for some alternatives for the treatment of deadly typhoid is imperative due to increasing MDR. The need is to explore/ extract active constituents from propolis and then use these in combination with standard antibiotics to reduce their doses and improve efficacy without developing MDR or any side effects.

## Abbreviations

ALP: Alkaline phosphatase
ALT: Alanine transaminase
AST: Aspartate transaminase
b.w: Body weight
CAPE: Caffeic acid phenyl ethyl ester
CFU: Colony forming units
DCT: Distal convoluted tubules
DLC: Differential leucocyte count
EDTA: Ethylenediaminetetraacetic acid
Hb: Haemoglobin
i.p.: Intraperitonial
MCH: Mean corpuscular haemoglobin
MCHC: Mean corpuscular haemoglobin concentration
MCV: Mean corpuscular volume
MDR: Multi Drug Resistance
PALS: Periarterial lymphatic sheaths
PCT: Proximal convoluted tubules
PCV: Packed cell volume
RBC: Red blood cell count
rpm: Rotations per minute
SGOT: Serum glutamate oxaloacetate transaminase
SGPT: Serum glutamate pyruvate transaminase
TLC: Total leucocyte count
