# Erratum to: Studies on the therapeutic effect of propolis along with standard antibacterial drug in *Salmonella enterica* serovar Typhimurium infected BALB/c mice

**DOI:** 10.1186/s12906-016-1523-0

**Published:** 2017-01-05

**Authors:** Preeti Kalia, Neelima R. Kumar, Kusum Harjai

**Affiliations:** 1 Department of Zoology, Panjab University, Chandigarh, India; 2 Department of Microbiology, Panjab University, Chandigarh, India

## Erratum

Following publication of the original article [1] it was brought to our attention that some incorrect superscripts had been added to the three bars for group CP4 in Fig. 1. Please therefore see below for the corrected figure:

**Fig. 1:**
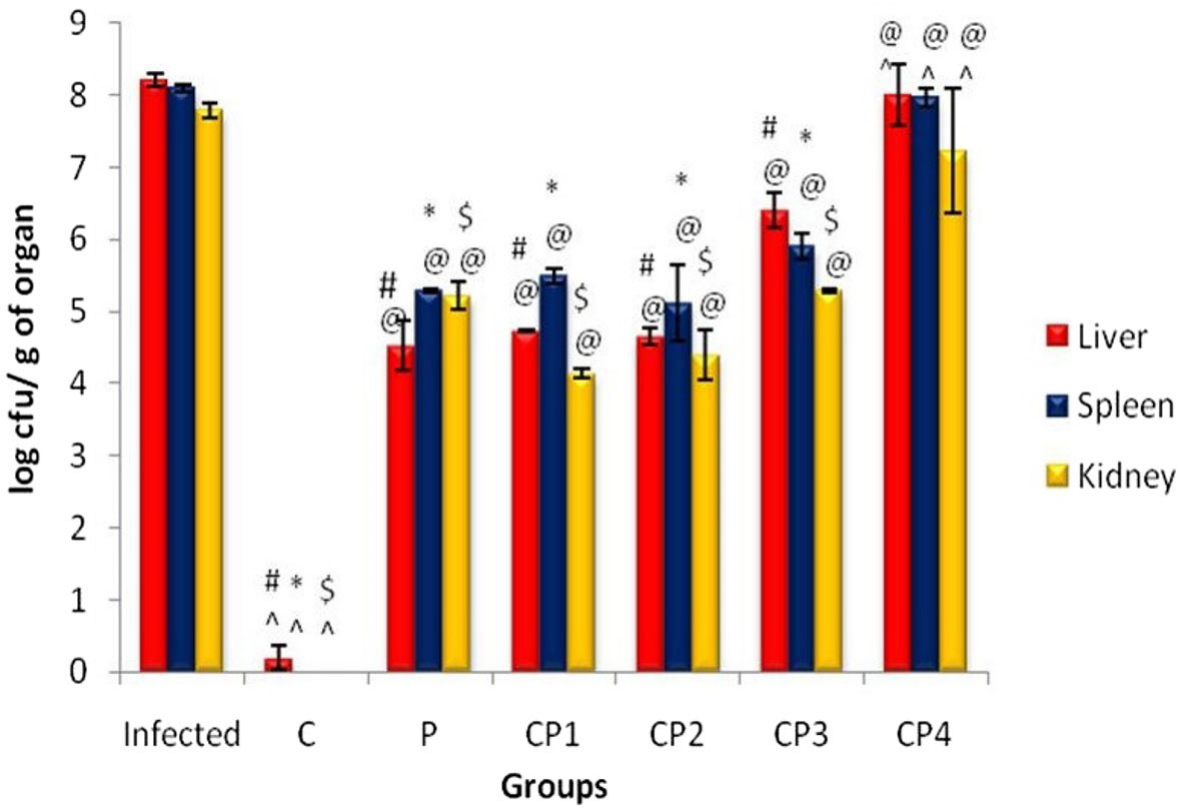
Histogram showing the bacterial load in different organs of mice after Salmonella enterica serovar Typhimurium infection and treatment in combination groups. Data is expressed as mean ± SD. ^#^ *p*-value Infected liver vs treated liver, *p-value Infected spleen vs treated spleen. ^$^ *p*-value Infected kidney vs treated kidney. ^@^ *p*-value C vs P, CP1, CP2, CP3 and CP4. ^*p*-value P vs C, CP1, CP2, CP3 and CP4. (^@^^*p* < 0.05), (^#^*^$^ *p*< 0.001)

Please note that the original article was corrected on the BioMed Central website.
